# Japanese encephalitis vaccination in the Philippines: A cost-effectiveness analysis comparing alternative delivery strategies

**DOI:** 10.1016/j.vaccine.2020.02.018

**Published:** 2020-03-17

**Authors:** Elisabeth Vodicka, Marita Zimmermann, Anna Lena Lopez, Maria Wilda Silva, Leonita Gorgolon, Toda Kohei, Jessica Mooney, Farzana Muhib, Clint Pecenka, Anthony A. Marfin

**Affiliations:** a PATH, 2201 Westlake Ave, Suite 200, Seattle, WA 98121, USA; b Consultants in Global Health, LLC, 1523B NW 64th St., Seattle, WA 98107, USA; c University of the Philippines Manila National Institutes of Health, Institute of Child Health and Human Development, 623 Pedro Gil Street, Ermita 1000, Manila, Philippines; d Department of Health, San Lazaro Compound, Rizal Ave., Sta. Cruz, Manila, Philippines; e World Health Organization, Regional Office for the Western Pacific, P.O. Box 2932, 1000 Manila, Philippines

**Keywords:** Japanese encephalitis, Japanese encephalitis vaccine, Cost-effectiveness, Cost analysis, Philippines

## Abstract

**Introduction:**

Japanese encephalitis (JE) is a mosquito-borne viral infection of the brain that can cause permanent brain damage and death. In the Philippines, efforts are underway to deliver a live attenuated JE vaccine (CD-JEV) to children under five years of age (YOA), who are disproportionately infected. Multiple vaccination strategies are being considered.

**Methods:**

We conducted a cost-effectiveness analysis comparing three vaccination strategies to the current state of no vaccination from the societal and government perspectives: (1) national routine vaccination only, (2) sub-national campaign followed by national routine, and (3) national campaign followed by national routine. We developed a Markov model to estimate impact of vaccination or no vaccination over the child’s lifetime horizon, assuming an annual incidence of 10.6 cases per 100,000.

Costs of illness ($859/case), vaccine ($0.50/dose), routine vaccination ($0.95/dose), and campaign vaccination ($0.98/dose) were based on hospital financial records, expert opinion and literature. The societal perspective included transportation and opportunity costs of caregiver time, in addition to costs incurred by the health system.

**Results:**

JE vaccination via national campaign followed by national routine delivery was the most cost-effective strategy modeled with a cost per disability adjusted life year (DALY) averted of $233 and $29 from the government and societal perspectives, respectively. Results were similar for other delivery strategies with cost/DALY ranging from $233 to $265 from the government perspective and $29–$57 from the societal perspective. JE vaccination was projected to prevent 27,856–37,277 cases, 5571–7455 deaths, and 173,233–230,704 DALYs among children under five over 20 consecutive birth cohorts. Total incremental costs of vaccination versus no vaccination over 20 birth cohorts were $6.6–$9.8 million from the societal perspective ($230 K–$440 K annually) and $45.9–$53.9 million ($2.2 M–$2.7 M annually) from the governmental perspective.

**Conclusion:**

Vaccination with CD-JEV in the Philippines is projected to be cost-effective, reducing long-term costs associated with JE illness and improving health outcomes compared to no vaccination.

## 1 Introduction

Japanese encephalitis (JE) is a mosquito-transmitted viral infection of the brain [1]. Although 99% of infected people do not develop symptoms, symptomatic JE kills up to 30% of those affected and disproportionately impacts children, leaving up to half of survivors with permanent brain damage such as cognitive impairment, paralysis, seizures, inability to speak, memory loss, and other mental disorders [2]. Several JE vaccines have been developed and are currently used in routine immunization in many JE-endemic Asian countries [3]. CD-JEV, a live attenuated JE vaccine with favorable safety and efficacy profiles, has been in use for over 30 years and is the least costly JE vaccine on the market. During the three decades of its use, over 400 million CD-JEV doses have been administered with few adverse events following immunization, most commonly injection site reactions and mild/moderate fever, vomiting or irritability [4], [5].

JE incidence has been significantly reduced in countries that have introduced CD-JEV. Previously high-incidence countries that have established JE vaccination programs (e.g., China, Japan, Republic of Korea) have achieved JE incidence rates as low as 0.003 per 100,000 [6], [7]. In contrast, high-incidence countries without JE vaccination programs (e.g., Philippines, Myanmar, Indonesia) have incidence rates of roughly 10 per 100,000 or greater [6]. In the Philippines, a recent systematic review reported that JE virus was the causative agent of 16–40% of all clinical encephalitis cases [8]. Regional variation exists in terms of both reported endemicity and surveillance capacity for identifying new JE cases at the regional level. According to the Philippines Department of Health (DoH) JE surveillance data from January 1 to December 31, 2018, the highest number of laboratory-confirmed JE cases were reported in the Ilocos (19% of JE cases), Cagayan Valley (13%), and Central Luzon (31%) Regions [9].

JE prevention efforts among children are underway in the Philippines. In 2008, a national surveillance program (Philippines Integrated Disease Surveillance and Response) was established to monitor cases of acute encephalitis syndrome to better understand JE disease burden in children and adults [8]. Additionally, the National Immunization Program (NIP) within the DoH is evaluating options for giving CD-JEV to children less than five-years-old. Introducing JE vaccine via one-time campaigns or through routine immunization programs can have a profound impact on health outcomes but can also incur substantial costs to the government. As a lower-middle income country, the Philippines government is responsible for self-financing JE vaccine introduction. While some Gavi-eligible countries have recently introduced the vaccine targeting children less than 15 years old [10], a target population of less than 5 years was considered more feasible for the Philippines given budgetary and health service delivery constraints in conducting large-scale campaigns. In addition to costs, more information is needed to identify an optimal delivery strategy considering key factors, such as financial and economic impact, healthcare resource utilization, and impact on health outcomes (e.g., JE prevention). To support decision-making for the national government, we conducted a cost-effectiveness analysis comparing potential JE vaccination via delivery strategies under consideration to the current state of no JE vaccination in the Philippines.

## 2 Materials and methods

In routine childhood immunization, JE vaccine is typically administered to children under 1 year old. However, since the vaccine is not currently available in the Philippines, a one-time campaign to vaccinate a broader age group of children in addition to routine immunization may be an opportunity to prevent additional JE disease burden in a population vulnerable to disease. We modeled three vaccination strategies currently under consideration by the DoH: (1) nationwide routine vaccination of 9-month-old children under the National Immunization Program (“National Routine”) in all 17 regions in the country; (2) a one-time nationwide campaign for children one to five years of age followed by nationwide routine vaccination in 9-month-old children (“National Campaign + National Routine”); and (3) a one-time campaign for one- to five-year-old children in three regions—Ilocos (Region I), Cagayan Valley (Region II), and Central Luzon (Region III)—followed by nationwide routine vaccination in 9-month-old children (“Subnational Campaign + National Routine”). Based on discussions with the DOH, Regions I, II, and III were selected as likely locations for a campaign because they report a high number of JE cases and have operational capacity to conduct a CD-JEV campaign alongside routine immunization [8].

### 2.1 Model framework

**Fig. 1:**
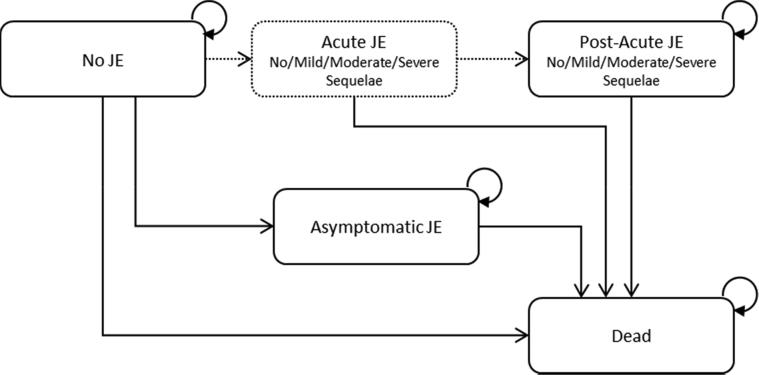
Markov model. All individuals enter the model with no JE. Acute JE implies symptomatic JE and is a tunnel state, meaning that any individual in that health state stays there for exactly one cycle. Those who had acute JE do have a higher mortality rate but must accrue the costs and DALYs of the acute event before transitioning to death. Asymptomatic JE is not associated with higher mortality, costs or DALYs; rather it eliminates any transition to acute JE. Costs and DALYs for acute and post-acute JE are distributed by sequelae presence and severity. Vaccination changes the probability of transitioning from no JE to acute or asymptomatic JE. No other probabilities are changed by presence or absence of vaccination. Each state is associated with an annual cost and disability weight where applicable.

We developed a Markov model to estimate the epidemiological and economic outcomes associated with JE vaccination of children five-years-old and younger compared to no vaccination. The model simulated children transitioning through five health states over the child’s lifetime horizon: no JE, acute JE, asymptomatic JE, post-acute JE, and death (Fig. 1). Children entered the model as healthy individuals in the “no JE” state and were assumed to be either vaccinated or not. Children could remain disease-free or transition to acute JE (i.e. symptomatic JE), asymptomatic JE infection, or death. Those who entered the acute JE state could have mild, moderate, or severe sequelae. Children in the acute JE state had a higher mortality rate and were assumed to accrue costs and quality-of-life decrements associated with this state. These individuals could not remain in the acute JE state, and they were modeled to transition to either post-acute JE or death. Asymptomatic JE was incorporated as a rate of asymptomatic JE to symptomatic JE, which is estimated to range from 25:1 to 1000:1. In the model, asymptomatic JE was not associated with any costs or change in health outcomes, as it was assumed to remain undetected and not require treatment. Further, children infected with either asymptomatic or symptomatic JE demonstrate natural immunity to future infections [6]. Therefore, in our model, children with asymptomatic JE could not transition to the symptomatic JE health state and those infected with symptomatic JE could not experience re-infection. All transitions between health states were modeled to occur over one-year cycles.

Health outcomes included the expected number of JE cases, deaths, and disability-adjusted life-years (DALYs) for vaccinated and unvaccinated cohorts under the different vaccination strategies. Life tables were used to calculate age-based background mortality rates for the Philippines, which were applied to each health state to capture non-JE deaths across the life cycle [11]. To incorporate potential impact on quality of life, disability weights were applied for acute JE for the duration of the health state and annually for each sequelae based on available global burden of disease estimates [12]. Vaccination was expected to affect the probability of transition from no JE to acute or asymptomatic JE. No other probabilities were changed by presence or absence of vaccination.

We assumed children receiving routine immunization would be vaccinated in the first year of life. For campaign immunization, we assumed that children would be vaccinated at the mid-point age of the target population (3 years old), although additional ages were evaluated through sensitivity analyses. Routine immunization was modeled over 20 birth cohorts of children, whereas the campaign was modeled as a one-time catch up. The annual crude birth rate (23.2 per 1000 people) was obtained from 2016 World Bank data [13]. Population sizes by region and age group were obtained from 2015 Philippines census data [14]. Additional model assumptions are outlined in Table 1. The base case analysis modeled JE vaccination from the government health system perspective. Secondary analysis was conducted from the societal perspective, in which we included opportunity costs of caregiver time and transportation costs for the patient and caregiver in addition to costs incurred by the health system. Additional categories of costs and outcomes included in each perspective are outlined explicitly in the Cost-Effectiveness Impact Inventory in Appendix Table 1 [15].

**Table 1:** Model assumptions for base case and scenario analyses.

**General assumptions for model framework**
• A single vaccination dose was administered with no booster.
• If vaccination was effective, JE protection remained over the rest of the individual’s lifetime [6].
• For those who experienced sequelae, the sequelae continued over the individual’s lifetime.
• Vaccination did not impact the duration or severity of JE if disease occurred.
• There were no serious adverse events associated with the JE vaccine.

**Model perspectives**
• Base Case: Government perspective • Secondary: Societal perspective

**Vaccination Strategies Evaluated for JE Vaccine Delivery**
1) JE vaccination delivered through national routine immunization over 20 cohorts of children vs. no vaccination 2) One-time national campaign followed by national routine immunization 20 cohorts of children vs. no vaccination 3) One-time subnational campaign in select regions followed by national routine immunization over 20 cohorts of children vs. no vaccination

Input parameters for JE epidemiology and vaccination are provided in Table 2. For the base case estimate, we used the annual incidence estimate for 0- to 15-year-old children living in high-incidence areas without JE vaccination programs (10.6 per 100,000) [6]; these estimates specifically included the Philippines. We estimated vaccine effectiveness (93%, range: 69–98%) using a pooled odds ratio and 95% confidence interval derived from results of five case-control studies evaluating CD-JEV effectiveness [3]. We assumed a vaccination coverage rate of 82% based on in-country reports of measles-containing-vaccine first-dose (MCV1) national immunization coverage in 2015 [16]. We assumed that CD-JEV was given as a one-dose primary and, when effective, conferred lifelong protection.

**Table 2:** Summary of model parameters for JE vaccination and epidemiology.

Parameter	Base case estimate	Range	Source
Acute/symptomatic JE incidence	10.6 per 100,000	8–13 per 100,000	[5]
Asymptomatic JE, incidence multiplier	300	25–1000	[27]
Case fatality (acute JE)	20%	10–30%	[2]
Duration of acute JE event	20 days	15.6–23.4 days	[28]
Sequelae incidence	40%	30%-50%	[6]

Sequelae severity			
Mild	48%	1-(Mod + Severe)	[29]
Moderate	24%	19%-29%	[29]
Severe	28%	22%-34%	[29]

Probability of treatment for sequelae			
Mild	0%	0	In-country expert opinion
Moderate	0%	0	In-country expert opinion
Severe	75%	60–90%	In-country expert opinion
Vaccine efficacy	93%	69–98%	[3]
Vaccine coverage	82%	66–98%	In-country expert opinion
Discount rate for costs and health outcomes	3%	0–5%	Assumption
Disability weight for acute JE (per event)	0.133	0.088–0.190	[12]Weight for “infectious disease acute episode – severe”
Disability weights for long term sequelae (annual)			
Mild	0.031	0.018–0.050	[12]Motor and cognitive impairments - mild
Moderate	0.203	0.134–0.290	[12]Motor and cognitive impairments - moderate
Severe	0.542	0.374–0.702	[12]Motor and cognitive impairments - severe

### 2.2 Resource use and costs

Primary data were collected to estimate costs of vaccine introduction and delivery, as well as costs of treatment for JE sequelae. In order to capture potential variation in vaccine delivery costs, JE-related treatment patterns, and resource utilization, these data were collected in two regions with large differences in the number of JE cases reported to the DoH – Region III (San Fernando, Pampanga) and Region XI (Davao). For example, in 2017, Region III reported more than 100 laboratory-confirmed JE cases compared to fewer than 20 such cases in Region XI. However, differences in case counts may reflect variation in established surveillance capacity rather than regional variation of JE incidence [5].

We estimated that the cost of vaccination delivery would be $0.50 per dose for the vaccine plus $0.95 or $0.98 per dose for operational costs for routine vaccination and campaign vaccination, respectively. Cost of vaccine delivery was based on expert opinion of key administrative and clinical personnel at the national and regional Departments of Health, facility-level and national laboratories, vaccine storage facilities, and the Western Pacific Regional Office of WHO. Since CD-JEV had not yet been introduced in the Philippines, total costs of implementing a JE vaccination program were estimated based on historical costs to the DoH of introducing previous vaccines for children at similar ages. Vaccination program cost components included expected expenditures on social mobilization, training, vaccine supplies, cold chain equipment, and personnel costs, among others (Table 3). Total program costs for implementing a one-time campaign at the regional level were estimated to range from $136,019 to $1,151,607, depending on total population to be covered (Appendix Table 2).

**Table 3:** Summary of model parameters for costs of JE vaccine delivery and JE treatment.

Parameter	Base case estimate range		Source
***JE treatment costs***			
Average length of stay (treatment days)	14	SE: 5.25	In country data collection
JE-Related Hospitalization Costs (per JE event)$859		$687–$2566	In country data collection
One-night stay/bed	$161	$129–$193	In country data collection
Provider time	$145	$116–$174	In country data collection
Medicine & Supplies	$305	$244–$306	In country data collection
Laboratory & X-Ray	$245	$196–$295	In country data collection
Other (e.g. oxygen, etc.)	$3	$2–$4	In country data collection
National disease surveillance costs	$5	$4–$6	In country data collection

Annual costs for sequelae			
Mild	$0	$0	Assumption
Moderate	$129	$103–$155	[30] Applied annual neurodevelopmental impairment cost
Severe	$143	$114–$171	[30] Applied annual epilepsy cost

***Vaccine-related costs***			
Vaccine cost to government (per dose)	$0.50	$0.40–$0.60	Assumption
Annual increase in vaccine cost	5%	4–6%	Assumption
Vaccine delivery cost per dose for routine immunization	$0.95	$0.76–$1.14	In country data collection
Vaccine delivery cost per dose for campaign	$0.98	$0.76–$1.14	In country data collection
Wastage	15%	12–18%	Assumption
Buffer	25%	20–30%	Assumption

***Patient and caregiver non-medical and indirect costs (included in societal perspective)***			
Patient transportation costs	$2.07	$1.66–$2.48	[15] Regional assumption
Caregiver transportation costs	$2.07	$1.66–$2.48	[15] Regional assumption
Caregiver meals during care	$31	$25–$37	[16] Regional assumption *Assumed 10% of caretaker expenses were meals.*
Caregiver lost wages due to time missed from work	$398	$318–$478	In country data collection. *Assumed average length of stay (14 days) with 1 day of caregiver time valued at GDP per capita/260 (number of working days), World Bank, recorded 2015*

The base case average cost of treatment was estimated at $859 (range: $687–$2566). To determine this cost of treatment, we conducted interviews with clinical, administrative, and laboratory staff at the main public hospital in the two regions as well as administrators at two private facilities to understand the potential magnitude of variation by facility type. Through these interviews, we identified the typical patient flow, length of stay for suspected JE, local treatment patterns, and direct costs of medical care associated with acute JE and sequelae (Table 3). The base case cost analysis relied on treatment costs based on financial reports for JE-suspected cases from facilities in Region III (26 suspected cases in 2017) and Region XI (14 suspected cases in 2015–2017). Total costs of patient visits for JE treatment were disaggregated into five components: medicines and supplies, lab and x-ray, room and board, professional fees, and other. Costs from facility records represent actual expenditures on JE-related care at public facilities in Regions III and XI.

Caregiver time was identified as a potentially important economic cost of treatment, as an adult is generally required to accompany a pediatric patient throughout each component of care, resulting in productivity losses associated with missed work or other activities. As such, we included opportunity costs of caregiver time in addition to travel costs in all analyses conducted from the societal perspective. We applied regional estimates from the literature [17], [18], [19] for transportation costs and non-medical costs. Lost wages of caregivers were based on local data on the average length of the child’s stay (two weeks) and the average daily wage, which was calculated using GDP over number of working days per year (Table 3). While we included health-related quality effects for children over their lifetime horizon with and without vaccination, we did not include spillover effects on health-related quality of life for caregivers.

For the cost-effectiveness model, we assumed that the cost of the vaccine would increase by 5% annually. We also assumed 15% and 10% vaccine wastage for routine and campaign strategies, respectively. A 25% vaccine buffer was included for routine immunization. A 3% discount rate was applied to all health outcomes and costs [15]. All costs were converted to 2017 US dollars [20].

### 2.3 Analytic methods

Health and cost outcomes were first calculated for an individual child from time of vaccination or no vaccination until death. For strategies involving a one-time campaign, individual results were summed for a cohort of children age 0–5 over their remaining lifetime across populations in regions included in the campaign. For routine immunization, the model aggregated outcomes for 20 birth cohorts (based on the current birth rate) over their remaining lifetime. For each introduction strategy evaluated, we projected the number of JE cases, life expectancy, disability-adjusted life-years, and deaths for vaccinated and unvaccinated populations simulated in the model. We then calculated Incremental Cost-Effectiveness Ratios (ICER), defined as incremental costs over incremental DALYs averted, as an estimate of the potential value achieved through each of the three vaccination strategies compared to no vaccination. We did not directly compare one vaccination strategy to another.

### 2.4 Uncertainty analyses

We conducted one-way sensitivity analyses to identify key drivers of model outcomes using available measures of parameter uncertainty (i.e. standard errors) or reasonable ranges for each input determined by the literature or expert opinion when magnitude of uncertainty was unknown. Key drivers were defined as parameters whose impact on model uncertainty was ≥15% of the total cost per DALY averted.

**Fig. 2:**
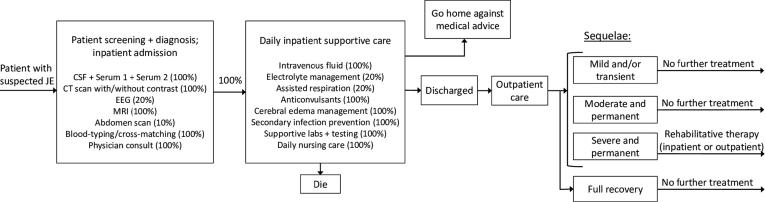
Typical patient flow for suspected acute JE in the Philippines^ⱡ^. *^ⱡ^This diagram represents comprehensive treatment for a typical patient entering a health care facility with suspected JE. The diagram was developed based on local expert opinion and iterated upon based on discussions with pediatric neurologists and clinicians in local health care facilities. Percentages included in the “Patient screening + diagnosis; inpatient admission” and “Daily inpatient supportive care” boxes represent the proportion of patients expected to receive the identified health care services. This patient flow map informed the secondary, unit-based costing exercise. Each component of the patient flow was assigned a cost based on unit data provided by facilities in Region III and Regional XI. A population-weighted cost including medicines, supplies, and staff time was applied to determine treatment costs relative to average length of patient stay. This cost represents the typical set of treatments we would expect for a patient with suspected JE without censoring that occurred in financial records when a patient transfers health care facilities. It was used as the high value for costs of treatment in the one-way sensitivity analysis.*

The base case costing analysis relied on cost estimates of treatment provided in two public hospitals. These estimates did not include costs of JE treatment received in other health centers before or after attending the public hospital. As such, we conducted a secondary costing exercise using a unit-based approach to estimate treatment costs based on the expected care cascade for a patient presenting with suspected JE, since this approach captures the full expected resource use in JE case treatment. For the exercise, expert opinion from discussions with pediatric neurologists and clinicians informed a patient care flow diagram depicted in Fig. 2. Each component of the patient flow was then assigned a cost based on unit data provided by facilities in Region III and Regional XI. A population-weighted cost including medicines, supplies, and staff time was applied to determine treatment costs relative to average length of stay. This cost represents the typical set of treatments we would expect for a patient with suspected JE without censoring and was used as the high value for costs of acute JE treatment in the one-way sensitivity analysis.

Probabilistic sensitivity analyses were also performed by jointly varying all model parameters over 10,000 Monte Carlo simulations to evaluate uncertainty. We used normal distributions for age, gender, odds ratios, population sizes, duration of JE, and asymptomatic JE; beta distributions for utilities and percentages; and gamma distributions for costs. Scenario analyses were conducted to evaluate the impact providing national routine immunization over 10 birth cohorts instead of 20 birth cohorts in order to identify the potential variation in budget impact and population health outcomes over varying time frames.

The model and uncertainty analyses were developed in Microsoft Excel™ (Redmond, WA).

## 3 Results

Clinical and economic outcomes for each potential vaccine delivery strategy over 20 birth cohorts are presented in Table 4. We estimated that national routine vaccination without any campaigns would prevent 27,856 JE cases, 173,233 DALYs, and 5,571 deaths compared to no vaccination. Campaigns in Regions I, II, and III only followed by national routine vaccination would prevent 29,687 cases, 184,403 DALYs, and 5,937 deaths compared to no vaccination. National campaigns in all regions followed by national routine vaccination would prevent 37,277 cases, 230,704 DALYs, and 7,455 deaths compared to no vaccination.

**Table 4:** Projected net costs and outcomes associated with routine and campaign JE vaccine under base case assumptions from the government payer and societal perspectives.

Incremental outcomes compared to no vaccination	National Routine		Subnational Campaign in Regions I, II, IIIⱡ + National Routine		National Campaign + National Routine	
	Government	Societal	Government	Societal	Government	Societal
Target population (N) ⱡⱡ	2,435,226		5,188,410		16,599,615	
# expected to be vaccinated annuallyⱡⱡ	1,996,885		4,254,497		13,611,684	
Cases averted	27,856		29,687		37,277	
DALYs averted	173,233		184,403		230,704	
Deaths averted	5571		5937		7455	
Total costsⱡⱡⱡ	$45.9 M	$9.8 M	$47.5 M	$9.2 M	$53.9 M	$6.6 M
Cost per case averted	$1651	$351	$1601	$309	$1445	$177
Cost per DALY averted	$265	$57	$258	$50	$233	$29
Cost per death averted	$8256	$1757	$8004	$1544	$7225	$884

ⱡ *Region I: Ilocos; Region II: Cagayan Valley; Region III: Central Luzon. Regions were selected based on number of cases reported and expected feasibility for conducting a JE-CDV catch-up campaign.*

ⱡⱡ *Target population represents the size of the birth cohort (for routine) or under 5 population (for campaign) to be targeted for vaccination. The # expected to be vaccinated annually represents target population multiplied by expected vaccine coverage rates under each scenario.*

ⱡⱡⱡ *Total costs refers to the projected incremental costs incurred by the government or society with each vaccination strategy compared to no vaccination over the population cohort’s lifetime horizon.*

For national routine vaccination without campaigns, we estimated total incremental costs over 20 birth cohorts of $45.9 million ($2.3 M annually) and $9.8 million from government and societal perspectives ($490 K annually), respectively, compared to no vaccination. For campaigns in three regions plus national routine vaccination over 20 birth cohorts, we estimated incremental costs of $47.5 million ($2.4 M annually) and $9.2 million ($460 K annually) from government and societal perspectives, respectively. Finally, for a national campaign followed by national routine vaccination, we estimated incremental costs of $53.9 million ($2.7 M annually) and $6.6 million ($330 K annually) from government and societal perspectives, respectively, compared to no vaccination.

National routine vaccination yielded an ICER of $265 per DALY averted from the government payer perspective and $57 per DALY averted from the societal perspective compared to no vaccination. A subnational campaign in Regions I, II, and III followed by national routine vaccination resulted in an ICER of $258 from the government perspective and $50 from the societal perspective compared to no vaccination. A national campaign followed by national routine vaccination resulted in an ICER of $233 from the government perspective and $29 from the societal perspective compared to no vaccination.

### 3.1 Uncertainty analysis

**Fig. 3:**
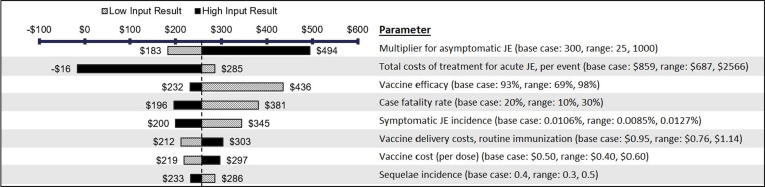
One-way sensitivity analysis of key cost drivers* for cost per DALY averted from the government perspective with Subnational Campaign + National Routine over 20 birth cohorts. *Key drivers were defined as parameters whose impact on model uncertainty was ≥15% of the total cost per DALY averted.

Results from the one-way sensitivity analysis are presented as a tornado diagram in Fig. 3 for the scenario of Subnational Campaign + National Routine. The rate of asymptomatic JE and cost of acute JE treatment were the most influential on model outcomes due to the wide uncertainty range for these parameters. Notably, when the high-range value for acute JE treatment costs is used ($2,566 from patient flow exercise) and all other parameters are held constant, JE vaccination is projected to be cost-saving. Other key drivers of the model included the rate of vaccine efficacy, case fatality rate, incidence of symptomatic JE, service delivery costs for routine immunization, vaccine cost per dose, and rate of severe sequelae. Key cost drivers were projected to be the same for the other two introduction strategies evaluated; however, the order of influence differs slightly for the National Campaign + National Routine strategy (see Supplementary Appendix Figs. 1 and 2).

Probabilistic sensitivity analyses suggest that the model is robust to uncertainty (see Supplementary Appendix Fig. 3). We also conducted scenario analyses to characterize how the model results might change when we modeled routine immunization over 10 birth cohorts instead of 20. Use of a smaller number of cohorts did not directionally change results but resulted in lower incurred costs and fewer health outcomes averted due to fewer children receiving vaccination (see Appendix Table 3).

## 4 Discussion

Introducing CD-JEV in the Philippines through routine and/or campaign-based vaccination strategies is projected to reduce mortality and morbidity compared to no vaccination with an increase in government expenditures. When the analysis was conducted from the societal perspective that included opportunity costs of caregivers’ time, the potential value of vaccination was amplified with the same improvements in health outcomes but lower incremental costs.

WHO recommends JE vaccination as a safe and effective measure to prevent child deaths in JE-endemic areas [1]. Our findings indicate that JE vaccination in the Philippines also provides good value for money. ICERs below the per-capita gross domestic product are often viewed favorably, although challenges with this threshold approach exist [21]. Our study found that vaccination would result in an ICER ranging from $29 to $265 depending on strategy and perspective. Compared to the GDP per capita in the Philippines ($2,951 in 2016), our modeled vaccination scenarios show DALYs averted at only 2–9% of the Philippines GDP per capita. ICERs in this range are historically viewed as good buys in global health [22], [23]; however, ICERs should be considered within the context of currently funded interventions in the absence of a nationally-established cost-effectiveness threshold [24]. Our model suggests that conducting a national campaign followed by national routine vaccination would be more cost-effective than routine vaccination alone or combined with subnational campaigns. However, these findings should be weighed against feasibility, budget implications and affordability given other investment priorities for the government [25].

These are the first estimates of JE vaccine introduction costs in the Philippines. They provide an understanding of the current health and economic burden of JE as well as the magnitude of potential benefits that disease prevention through vaccination may yield. To our knowledge, the assumptions used in our study reflect the current epidemiologic information and best available data for the Philippines. Though our cost estimates were collected from a limited sample of facilities, they were comparable to estimates from other Asian countries. Cost-effectiveness studies of JE immunization programs in China [26], [27], Indonesia [18], and Cambodia [28] estimated the total cost of vaccine and delivery to be $1.15, $0.50, $1.80-$2.30, and $2.41 per child per dose, respectively. Our estimates of $1.45 (routine) and $1.48 (campaign) per child per dose are comparable. The prior studies also estimated acute JE care costs ranging from $350 to $1,209 per case in constant 2017 US dollars, which is in line with our base case estimates of $859 per case. Our economic findings were similar to findings reported from studies in China, Indonesia, and Cambodia that projected that vaccination would improve health outcomes while incurring moderate incremental costs compared to no vaccination.

The ICERs elicited from our analysis are somewhat higher than those reported in prior studies that identified costs per DALY of $22 to $96 (original dollar values) [18], [27], [28]. These studies incorporated different underlying model assumptions in key parameters, including analytic perspective (i.e., whether caregiver non-medical and/or indirect costs were included), incidence rates, vaccine coverage and efficacy, number of vaccine doses, and treatment costs. For example, all studies assumed higher vaccine coverage ranging from 85 to 98% per dose and most assumed a two-dose strategy. In contrast, we applied an 82% coverage estimate assuming a one-dose strategy, which may be more realistic in the Philippines due to geographic and uptake considerations related to vaccination. However, changes in coverage estimates were not expected to significantly impact results. We also assumed a lower vaccine efficacy based on a pooled estimate of 93% compared to ≥95% applied in the other studies. A higher efficacy (98%) would make vaccination more favorable compared to no vaccination ($232/DALY averted), whereas lower efficacy (69%) would make vaccination less favorable ($436/DALY averted). These parameters all contribute to different costs per DALY averted between our study and the literature.

Some limitations to our study exist. First, minimal data is available on age-specific estimates of JE incidence in the Philippines. While surveillance programs to monitor acute meningitis/encephalitis syndrome cases exist and are steadily improving, reporting has often been inconsistent. As such, we applied a pooled estimate for highly endemic Asian regions, including the Philippines, without vaccination programs [6]. We further did not include adverse events in the model, although most are mild/moderate and not likely to result in significant costs or health outcomes. Second, we were limited in our ability to identify costs of treatment for confirmed JE. Instead, we estimated costs of treating acute encephalitis syndrome. This may be over- or underestimating the costs of treatment for confirmed JE cases; however, we expect that treatment strategies for JE and similar diseases such as acute encephalitis or meningitis would be comparable. Importantly, we were unable to capture the full economic impact of the vaccination program on society. For example, we did not include future unrelated medical costs, non-health care sector costs or spillover health-related quality of life effects for caregivers [15], [29]. Further, cost of delivery estimates did not capture the full breadth of economic costs associated with some vaccine delivery programs, such as donated equipment or the value of volunteer staff, as these were not identified in our data collection process but may be relevant for cost-effectiveness analyses of JE in other settings.

For opportunity costs of caregiver time, we calculated caregiver daily wage using GDP per capita divided by number of workdays, which may result in a potentially high estimate. Finally, a patient’s full length of stay may not be captured by our hospital-based estimates, since individuals in the Philippines often initiate treatment in more costly private facilities and transfer into public facilities due to financial challenges. While we were unable to capture full costs incurred across facilities due to censoring, we conducted a secondary analysis based on typical patient flow that includes expected costs over an expected full course of treatment to use as the high estimate of our one-way sensitivity analysis. These patient flow costs were much higher than the costs estimated from financial records ($2,566 vs. $859). When a higher cost of treatment was assumed, JE vaccination became cost-saving. This may be of particular interest to the Philippines and similar countries that are transitioning from lower-middle-income to upper-middle-income. In these countries, health care and rehabilitation costs may be increasing yet JE endemicity remains high, a combination that can greatly augment the value of JE vaccination.

In general, implementing routine JE vaccination with or without campaigns is expected to generate good value for money in the Philippines by improving health outcomes for relatively low incremental costs. Still, decision-makers must consider affordability and the cost of JE vaccine introduction which needs to be weighed against other population health needs. However, the demonstrated vaccine efficacy and safety combined with projected economic outcomes suggest that introduction and delivery of CD-JEV would reduce the health and economic burden of JE in the Philippines.

## Funding

This work was supported by the Bill & Melinda Gates Foundation, Seattle, WA [grant number OPP1115522].

## CRediT authorship contribution statement

**Elisabeth Vodicka:** Methodology, Formal analysis, Validation, Investigation, Visualization, Writing - original draft, Writing - review & editing. **Marita Zimmermann:** Conceptualization, Methodology, Formal analysis, Writing - review & editing. **Anna Lena Lopez:** Conceptualization, Methodology, Validation, Resources, Writing - review & editing. **Maria Wilda Silva:** Methodology, Validation, Resources, Writing - review & editing. **Leonita Gorgolon:** Validation, Resources, Writing - review & editing. **Toda Kohei:** Validation, Resources, Writing - review & editing. **Jessica Mooney:** Conceptualization, Project administration, Methodology, Investigation, Writing - review & editing. **Farzana Muhib:** Conceptualization, Validation, Writing - review & editing. **Clint Pecenka:** Conceptualization, Supervision, Validation, Writing - review & editing. **Anthony A. Marfin:** Supervision, Methodology, Validation, Writing - review & editing, Funding acquisition.

## Declaration of Competing Interest

The authors declare that they have no known competing financial interests or personal relationships that could have appeared to influence the work reported in this paper.

## References

[1] Japanese Encephalitis Vaccines (2016). WHO position paper, February 2015 - Recommendations. Vaccine.

[2] Solomon T., Dung N.M., Kneen R., Gainsborough M., Vaughn D.W., Khanh V.T. (2000). Japanese encephalitis. J Neurol Neurosurg Psychiatry.

[3] Li X., Ma S.J., Liu X., Jiang L.N., Zhou J.H., Xiong Y.Q. (2014). Immunogenicity and safety of currently available Japanese encephalitis vaccines: A systematic review. Hum Vaccines Immunother.

[4] PATH. Safety of CD-JEV: A proven tool for JE prevention; 2018. https://path.azureedge.net/media/documents/CVIA_Safety_CD_JEV_fs.pdf [accessed January 20, 2019].

[5] WHO (2016). Information sheet observed rate of vaccine reactions - Japanese encephalitis vaccine. Inf Sheet.

[6] Campbell G., Hills S., Fischer M., Jacobson J., Hoke C., Hombach J. (2011). Estimated global incidence of Japanese encephalitis: a systematic review. Bull World Health Organ.

[7] Tsai T.F. (2000). New initiatives for the control of Japanese encephalitis by vaccination. Vaccine.

[8] Lopez A.L., Aldaba J.G., Roque V.G., Tandoc A.O., Sy A.K., Espino F.E. (2015). Epidemiology of Japanese encephalitis in the Philippines: a systematic review. PLoS Negl Trop Dis.

[9] Republic of the Philippines Department of Health. Japanese Encephalitis Monthly Surveillance Report No. 12; 2018.

[10] Heffelfinger J.D., Li X., Batmunkh N. (2017). Japanese encephalitis surveillance and immunization — Asia and Western Pacific Regions, 2016. MMWR Morb Mortal Wkly Rep.

[11] Life tables by country - Philippines. World Health Organization. Global Health Observatory Data Repository; 2015. http://apps.who.int/gho/data/view.main.LT62150?lang=en [accessed March 1, 2017].

[12] Salomon J.A., Haagsma J.A., Davis A., de Noordhout C.M., Polinder S., Havelaar A.H. (2015). Disability weights for the Global Burden of Disease 2013 study. Lancet Glob Heal.

[13] The World Bank. Birth rate, crude (per 1000 people). Data from Database Popul Estim Proj Last Updat Sept 20, 2018 Available Online Http//DatabankWorldbankOrg/Data/Source/Population-Estimates-and-Projections# [last accessed Novemb 12, 2018 n.d.].

[14] Philippine Statistics Authority, 2015 Census of Population. Accessed online at: http://psa.gov.ph/content/highlights-philippine-population-2015-census-population. n.d.

[15] Sanders G.D., Neumann P.J., Basu A., Brock D.W., Feeny D., Krahn M. (2016). Recommendations for conduct, methodological practices, and reporting of cost-effectiveness analyses: second panel on cost-effectiveness in health and medicine. JAMA - J Am Med Assoc.

[16] Department of Health Internal Report; unpublished. MCV1 Vaccine Coverage, Philippines. 2015.

[17] Kim S.Y., Sweet S., Slichter D., Goldie S.J. (2010). Health and economic impact of rotavirus vaccination in GAVI-eligible countries. BMC Public Health.

[18] Liu W., Clemens J.D., Kari K., Xu Z.-Y. (2008). Cost-effectiveness of Japanese encephalitis (JE) immunization in Bali, Indonesia. Vaccine.

[19] The World Bank. “GDP per Capita.” https://data.worldbank.org/indicator/NY.GDP.PCAP.CD [accessed April 30, 2018. n.d.].

[20] World Economic Outlook Data (IMF; Oct 2017); https://www.exchange-rates.org/history/PHP/USD/T n.d.

[21] Bertram M.Y., Lauer J.A., De Joncheere K., Edejer T., Hutubessy R., Kieny M.-P. (2016). Cost–effectiveness thresholds: pros and cons. Bull World Health Organ.

[22] Hutubessy R, Chisholm D, Edejer TT-T, WHO-CHOICE. Generalized cost-effectiveness analysis for national-level priority-setting in the health sector. Cost Eff Resour Alloc 2003;1:8. doi:10.1186/1478-7547-1-8. 10.1186/1478-7547-1-8 PMC320499 14687420

[23] World Bank (1993). World development report 1993: Investing in health.

[24] Leech A.A., Kim D.D., Cohen J.T., Neumann P.J. (2018). Use and misuse of cost-effectiveness analysis thresholds in low- and middle-income countries: trends in cost-per-DALY studies. Value Heal.

[25] Bilinski A., Neumann P., Cohen J., Thorat T., McDaniel K., Salomon J.A. (2017). When cost-effective interventions are unaffordable: Integrating cost-effectiveness and budget impact in priority setting for global health programs. PLOS Med.

[26] Ding D., Kilgore P.E., Clemens J.D., Wei L., Zhi-Yi X. (2003). Cost-effectiveness of routine immunization to control Japanese encephalitis in Shanghai, China. Bull World Health Organ.

[27] Yin Z., Beeler Asay G.R., Zhang L., Li Y., Zuo S., Hutin Y.J. (2012). An economic evaluation of the use of Japanese encephalitis vaccine in the expanded program of immunization of Guizhou province, China. Vaccine.

[28] Touch S., Suraratdecha C., Samnang C., Heng S., Gazley L., Huch C. (2010). A cost–effectiveness analysis of Japanese encephalitis vaccine in Cambodia. Vaccine.

[29] Garrison L.P., Mansley E.C., Abbott T.A., Bresnahan B.W., Hay J.W., Smeeding J. (2010). Good research practices for measuring drug costs in cost-effectiveness analyses: A societal perspective. The ISPOR drug cost task force report - Part II. Value Heal.

[30] Haasis M.A., Ceria J.A., Kulpeng W., Teerawattananon Y., Alejandria M. (2015). Do Pneumococcal Conjugate Vaccines Represent Good Value for Money in a Lower-Middle Income Country? A Cost-Utility Analysis in the Philippines.. PLoS One.

