# Supplementary material for: Japanese encephalitis vaccination in the Philippines: A cost-effectiveness analysis comparing alternative delivery strategies
Source: Vaccine. 2020 Mar 17;38(13):2833–40. doi: 10.1016/j.vaccine.2020.02.018 (PMC7068699; doi:10.1016/j.vaccine.2020.02.018)
Supplement: Supplementary data 1 [file mmc1.docx]

**Supplementary Materials**

Appendix Table 1. Cost-Effectiveness Impact Inventory demonstrating types of health outcomes and costs included in each perspective

| Sector | Type of Impact | Included in analysis from the following perspective? | |
| --- | --- | --- | --- |
|  | *(Categories impacted within each sector with unit of measure if relevant)* | *Government* | *Societal* |
| Health | *Health Outcomes (effects)* |  |  |
|  | Longevity effects, expected life years | 🗸 | 🗸 |
|  | Health-related quality of life effects, DALYs | 🗸 | 🗸 |
|  | Other health effects (AEs, secondary infections, etc.) | 🗸 | 🗸 |
|  | Spillover health-related quality of life effects, caregiver | 🗴 | 🗴 |
|  |  |  |  |
|  | *Medical Costs* |  |  |
|  | Direct medical costs | 🗸 | 🗸 |
|  | Future related medical costs | 🗸 | 🗸 |
|  | Future unrelated medical costs | 🗴 | 🗴 |
|  |  |  |  |
| Health | Patient time costs | NA | 🗸 |
|  | Unpaid caregiver time costs | NA | 🗸 |
|  | Transportation costs | NA | 🗸 |
| Productivity | Cost of unpaid lost productivity due to illness | NA | 🗸 |
| Consumption | None | NA | 🗴 |
| Social Services | None | NA | 🗴 |
| Legal/Criminal Justice | None | NA | 🗴 |
| Education | None | NA | 🗴 |
| Housing | None | NA | 🗴 |
| Environment | None | NA | 🗴 |

Appendix Table 2. Incremental costs and outcomes of a one-time campaign for CD-JEV from the government perspective by region

|  | ***Annual Target Population*** | | ***One-Time Vaccination Campaign*** | | | |
| --- | --- | --- | --- | --- | --- | --- |
|  | **Annual under 5 population** | **# vaccinated annually** | **Cases averted** | **Deaths averted** | **DALYs averted** | **Incremental Costs** |
| *Region I (Ilocos)* | 698,423 | 572,707 | 465 | 93 | 2,834 | 388,141 |
| *Region II (Cayagan Valley)* | 479,484 | 393,177 | 319 | 64 | 1,945 | 266,468 |
| *Region III (Central Luzon)* | 1,575,277 | 1,291,727 | 1,048 | 210 | 6,392 | 875,442 |
| *Region IV-A (Calabarzon)* | 2,072,210 | 1,699,212 | 1,378 | 276 | 8,408 | 1,151,607 |
| *Region IV-B (Mimaropa)* | 417,517 | 342,364 | 278 | 56 | 1,694 | 232,030 |
| *Region V (Bicol)* | 808,592 | 663,046 | 538 | 108 | 3,281 | 449,366 |
| *Region VI (Western Visayas)* | 1,052,119 | 862,738 | 700 | 140 | 4,269 | 584,703 |
| *Region VII (Central Visayas)* | 1,033,286 | 847,294 | 687 | 137 | 4,193 | 574,237 |
| *Region VIII (Eastern Visayas)* | 605,075 | 496,162 | 402 | 80 | 2,455 | 336,263 |
| *Region IX (Zamboanga Peninsula)* | 520,921 | 427,155 | 346 | 69 | 2,114 | 289,496 |
| *Region X (Northern Mindanao)* | 664,496 | 544,887 | 442 | 88 | 2,696 | 369,286 |
| *Region XI (Davao)* | 687,407 | 563,674 | 457 | 91 | 2,789 | 382,018 |
| *Region XII (Soccsksargen)* | 651,254 | 534,028 | 433 | 87 | 2,642 | 361,927 |
| *Cordillera Administrative Region* | 244,754 | 200,698 | 163 | 33 | 993 | 136,019 |
| *Autonomous Region in Muslim Mindanao* | 487,132 | 399,448 | 324 | 65 | 1,977 | 270,718 |
| *Caraga Administrative Region* | 362,933 | 297,605 | 241 | 48 | 1,473 | 201,696 |
| *National Capital Region* | 1,803,509 | 1,478,877 | 1,200 | 240 | 7,318 | 1,002,279 |
| ***Total*** | **14,164,389** | **11,614,799** | **9,421** | **1,885** | **57,473** | **$7,871,696** |

Appendix Table 3. Incremental costs and outcomes of CD-JEV routine immunization delivery projected over 10 and 20 birth cohorts from the government
perspective by region

|  | ***Annual Target Population*** | | ***Routine Vaccination over 10 Birth Cohorts*** | | | | ***Routine Vaccination over 20 Birth Cohorts*** | | | |
| --- | --- | --- | --- | --- | --- | --- | --- | --- | --- | --- |
|  | **Annual birth cohort** | **# vaccinated annually** | **Cases averted** | **Deaths averted** | **DALYs averted** | **Incremental Costs** | **Cases averted** | **Deaths averted** | **DALYs averted** | **Incremental Costs** |
| *Region I (Ilocos)* | 120,077 | 98,463 | 788 | 158 | 4,898 | $1,092,931 | 1,374 | 275 | 8,542 | $2,267,837 |
| *Region II (Cayagan Valley)* | 82,436 | 67,597 | 541 | 108 | 3,362 | $750,323 | 943 | 189 | 5,864 | $1,556,923 |
| *Region III (Central Luzon)* | 270,831 | 222,081 | 1,776 | 355 | 11,046 | $2,465,078 | 3,098 | 620 | 19,266 | $5,115,051 |
| *Region IV-A (Calabarzon)* | 356,267 | 292,139 | 2,337 | 467 | 14,531 | $3,242,706 | 4,075 | 815 | 25,343 | $6,728,631 |
| *Region IV-B (Mimaropa)* | 71,782 | 58,861 | 471 | 94 | 2,928 | $653,353 | 821 | 164 | 5,106 | $1,355,710 |
| *Region V (Bicol)* | 139,018 | 113,995 | 912 | 182 | 5,670 | $1,265,329 | 1,590 | 318 | 9,889 | $2,625,564 |
| *Region VI (Western Visayas)* | 180,887 | 148,327 | 1,186 | 237 | 7,378 | $1,646,412 | 2,069 | 414 | 12,868 | $3,416,314 |
| *Region VII (Central Visayas)* | 177,649 | 145,672 | 1,165 | 233 | 7,246 | $1,616,941 | 2,032 | 406 | 12,637 | $3,355,161 |
| *Region VIII (Eastern Visayas)* | 104,028 | 85,303 | 682 | 136 | 4,243 | $946,854 | 1,190 | 238 | 7,400 | $1,964,727 |
| *Region IX (Zamboanga Peninsula)* | 89,560 | 73,439 | 587 | 117 | 3,653 | $815,164 | 1,024 | 205 | 6,371 | $1,691,471 |
| *Region X (Northern Mindanao)* | 114,244 | 93,680 | 749 | 150 | 4,660 | $1,039,840 | 1,307 | 261 | 8,127 | $2,157,673 |
| *Region XI (Davao)* | 118,183 | 96,910 | 775 | 155 | 4,820 | $1,075,691 | 1,352 | 270 | 8407 | $2,232,065 |
| *Region XII (Soccsksargen)* | 111,967 | 91,813 | 734 | 147 | 4,567 | $1,019,118 | 1,281 | 256 | 7,965 | $2,114,674 |
| *Cordillera Administrative Region* | 42,080 | 34,505 | 276 | 55 | 1,716 | $383,004 | 481 | 96 | 2,993 | $794,736 |
| *Autonomous Region in Muslim Mindanao* | 83,751 | 68,675 | 549 | 110 | 3,416 | $762,290 | 958 | 192 | 5,958 | $1,581,755 |
| *Caraga Administrative Region* | 62,398 | 51,166 | 409 | 82 | 2,545 | $567,937 | 714 | 143 | 4,439 | $1,178,473 |
| *National Capital Region* | 310,070 | 254,257 | 2,034 | 407 | 12,647 | $2,822,228 | 3,547 | 709 | 22,057 | $5,856,138 |
| ***Total*** | **2,435,228** | **1,996,883** | **15,971** | **3,193** | **99,326** | **$22,165,199** | **27,856** | **5,571** | **173,232** | **$45,992,903** |

Appendix Figure 1. One-way sensitivity analysis of key cost drivers* for cost per DALY averted from the government perspective with National Routine only over 20 birth cohorts

*
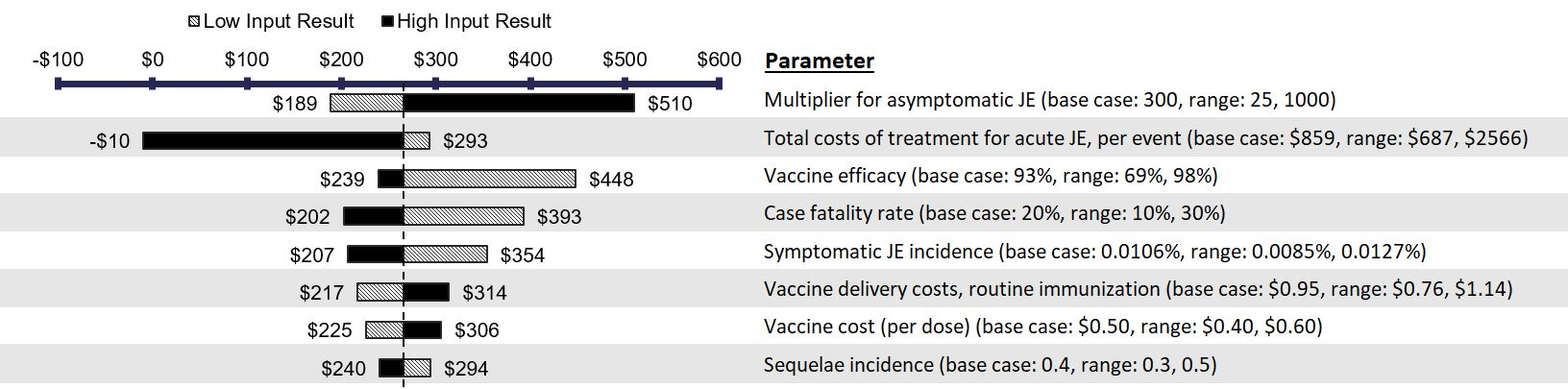

*Key drivers were defined as parameters whose impact on model uncertainty was ≥15% of the total cost per DALY averted*

Appendix Figure 2. One-way sensitivity analysis of key cost drivers* for cost per DALY averted from the government perspective with National Campaign + National Routine over 20 birth cohorts


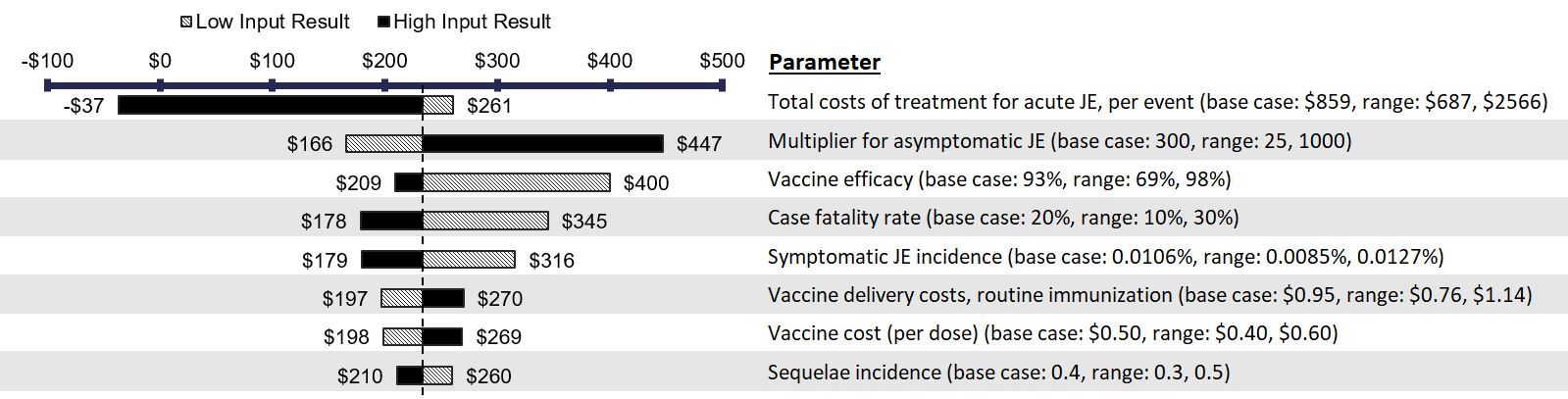
 **Key drivers were defined as parameters whose impact on model uncertainty was ≥15% of the total cost per DALY averted*

Appendix Figure 3. Probabilistic Sensitivity Analyses

Panel A. Incremental costs and DALYs averted with Subnational Campaign + National Routine immunization over 20 cohorts from the government perspective

Panel B. National Campaign + National Routine Immunization over 20 Cohorts from Government Perspective

Panel C. National Routine Immunization Only over 20 Cohorts from the Government Perspective
